# Gut Microbiota Dynamics during Dietary Shift in Eastern African Cichlid Fishes

**DOI:** 10.1371/journal.pone.0127462

**Published:** 2015-05-15

**Authors:** Laura Baldo, Joan Lluís Riera, Ave Tooming-Klunderud, M. Mar Albà, Walter Salzburger

**Affiliations:** 1 Zoological Institute, University of Basel, Basel, Switzerland; 2 Ecology Department, University of Barcelona, Barcelona, Spain; 3 University of Oslo, Centre for Ecological and Evolutionary Synthesis, Oslo, Norway; 4 Evolutionary Genomics Group, Institut Hospital del Mar d’Investigacions Mèdiques (IMIM), Barcelona, Spain; 5 University Pompeu Fabra (UPF), Barcelona, Spain; 6 Catalan Institution for Research and Advanced Studies (ICREA), Barcelona, Spain; Consiglio Nazionale delle Ricerche (CNR), ITALY

## Abstract

The gut microbiota structure reflects both a host phylogenetic history and a signature of adaptation to the host ecological, mainly trophic niches. African cichlid fishes, with their array of closely related species that underwent a rapid dietary niche radiation, offer a particularly interesting system to explore the relative contribution of these two factors in nature. Here we surveyed the host intra- and interspecific natural variation of the gut microbiota of five cichlid species from the monophyletic tribe Perissodini of lake Tanganyika, whose members transitioned from being zooplanktivorous to feeding primarily on fish scales. The outgroup riverine species *Astatotilapia burtoni*, largely omnivorous, was also included in the study. Fusobacteria, Firmicutes and Proteobacteria represented the dominant components in the gut microbiota of all 30 specimens analysed according to two distinct 16S rRNA markers. All members of the Perissodini tribe showed a homogenous pattern of microbial alpha and beta diversities, with no significant qualitative differences, despite changes in diet. The recent diet shift between zooplantkon- and scale-eaters simply reflects on a significant enrichment of *Clostridium* taxa in scale-eaters where they might be involved in the scale metabolism. Comparison with the omnivorous species *A*. *burtoni* suggests that, with increased host phylogenetic distance and/or increasing herbivory, the gut microbiota begins differentiating also at qualitative level. The cichlids show presence of a large conserved core of taxa and a small set of core OTUs (average 13–15%), remarkably stable also in captivity, and putatively favoured by both restricted microbial transmission among related hosts (putatively enhanced by mouthbrooding behavior) and common host constraints. This study sets the basis for a future large-scale investigation of the gut microbiota of cichlids and its adaptation in the process of the host adaptive radiation.

## Introduction

The gastrointestinal tract is home to an abundant and highly diverse community of microbes (i.e. the gut microbiota) that have evolved important nutritional and physiological dependencies among them and with the host [1–4]. Because a poorly functional gut microbiota can be detrimental to the host survival and fitness [5, 6], this internal ecosystem is emerging as a “novel” trait under strong natural selection [7], and a burst of studies is now attempting to understand its evolutionary dynamics with the host. On the one side, we expect the host to exert constraints in favour of a stable and long-term relationship with the microbiota, following the establishment of interdependencies (e.g. nutritional) that promote specialization and co-evolution of this symbiosis [3]. Indeed signature of host constraints has been detected in the microbiota of several animal systems, including bears [8], great apes [9], bat families [10] and fishes [11–13], and it is putatively favoured and maintained by a certain level of microbiota vertical transmission through host generations [14]. On the other side, the dynamic forces following ecological changes, especially diet, are expected to promote microbiota diversification for optimal and rapid adaptation to distinct ecological niches, as testified by microbiota divergence in related hosts with distinct diets or microbiota convergence in distant hosts within a trophic level [10, 15–18]. The actual role of host constraints and ecological pressures in shaping the gut microbiota remains, however, a matter of debate.

Comparative gut microbiomics of closely related species that underwent a rapid differentiation of ecological niches driven by diet (e.g. in adaptive radiations) provide an interesting approach to shed light on the microbiota dynamics at short evolutionary timescales (following speciation) and in response to both strong host genotypic effect and concurrent selective pressures for rapid adaptation to a trophic niche. While invertebrates have been extensively studied in this respect, research on vertebrate systems of closely related species still lags behind, although it has recently begun to flourish [10, 19].

Fishes are the most abundant and species-rich vertebrates and show incredible arrays of closely related species with a large spectrum of dietary niches; nevertheless they remain poorly studied in the wild and in the context of multiple related species [20–23]. Available microbiota research has primarily focused on single species, such as model systems, i.e. zebrafish [24], stickleback [25, 26], and the trinidian guppy *Peocilia reticulata* [27], or species relevant to aquaculture research: carp [28], atlantic salmon [29], turbot [4], atlantic cod [30] and the rainbow trout [31], among others (reviewed by [22]). Comparative microbiota studies across multiple fish species are rapidly increasing, yet to date they have primarily looked at unrelated species (e.g. asian carp and gizzard shad [32]; sticklebacks and perch [25]; notothenioid fishes [33]) or related fishes in captivity (i.e. Nicaraguan cichlids [34]), which have poorly informed on the”natural” state of the host-microbiota partnership.

Cichlid fishes from the explosive adaptive radiations in the three Eastern Africa Great lakes (Victoria, Malawi and Tanganyika) [35] have largely diversified following adaptation to distinct trophic niches, some as specialists (e.g. feeding on fish eyes or scales), others as generalist feeders [36]. Such diet radiation represented a main drive in the process of ecological speciation of this group, as testified by the extensive diversity of their cranial, jaw, teeth and intestine morphologies, coupled with their feeding strategy [35–37].

Lake Tanganyika is the oldest of the three major lakes and carries around 200 species, mostly endemic, which are currently subdivided into 14 tribes [36, 38]. One of these tribes, the Perissodini, has evolved a unique feeding habit primarily based on scales of other fishes (known as lepidophagy), which makes this tribe perhaps the most specialized among cichlids [39]. Perissodini represent a relatively young monophyletic clade of nine species (1.5–3.1 Ma [40, 41]), which transitioned from a mostly zooplanktivorous feeding habit (species belonging to the genus *Haplotaxodon*) to a scale-eating habit (species belonging to the genera *Plecodus* and *Perissodus*) following adaptation of feeding morphology (i.e. jaw and teeth) and behavior [36, 40, 42]. The scale-eating habit appears to have evolved once within this tribe [42] and proportion of scales in the diet has then progressively increased, with some species (e.g. *Perissodus microlepis* and *P*. *eccentricus*) feeding almost exclusively on scales. Scale-eaters mostly feed on scales of other cichlids, known as ctenoid scales: these are incredibly resistant structures largely made of collagen and covered by a bony layer [43]. Hence, scale-eaters have an unusually collagen-rich diet compared to zooplankton feeders, which might have led to a specialized digestive system and associated microbial communities.

In this study we profiled the composition of the gut microbiota of a sample of five of the nine recognized species within the tribe Perissodini, including both zooplankton- and scale-eaters. We also included the riverine omnivorous species *Astatotilapia burtoni*, belonging to the more distant tribe Tropheini, for which we characterized both a wild and a laboratory-inbred population. According to stable isotopes and gut content, *A*. *burtoni* is omnivorous, but largely feeds on algae and plants, making it partially “herbivorous” [36].

The main goals of this study were to 1) provide the first characterization of the African cichlid gut microbiota, 2) determine their core microbiota, and 3) explore the compositional dynamics of the microbiota as a function of both the diet shift and host phylogenetic constraints. We used two 16S fragments as markers and surveyed both intra- and inter-specific diversity, which allowed an unprecedented level of comparative analyses of both qualitative and quantitative gut microbiota profiles.

## Materials and Methods

### Sample collection

In June and July 2011 we sampled five cichlid species belonging to the tribe Perissodini from wild populations of the southern tip of lake Tanganyika (at the border between Zambia and Tanzania): the two zooplanktivorous species *Haplotaxodon microlepis* (designated as *Hapmic*, following [36]), and *H*. *trifasciatus* (*Haptri*); and the three scale-eaters, *Plecodus straeleni* (*Plestr)*, *Perissodus microlepis* (*Permic*) and *P*. *eccentricus* (*Perecc*) (S1 Table). The two shallow-water *Haplotaxodon* species largely feed on the pelagic shrimp *Mysis sp*. and partly on planktonic algae and crustaceans, diatoms and cupeid fry. *P*. *microlepis* and *P*. *straeleni* are the two most common scale-eaters in the lake and coexist in shallow rocky habitats while *P*. *eccentricus* inhabits deep waters. *P*. *microlepis* and *P*. *eccentricus* feed almost exclusively on scales, and occasionally skin tissues; in *P*. *straeleni* scales account for approximately 90% of its diet, integrated by fish skin and fry (for details on gut content and stable isotopes see [42] and [36]). A schematic host phylogenetic tree is represented in S1 Fig

We also sampled a member of the tribe Tropheini, *A*. *burtoni*, a species from an inflow river, including both a wild and an inbreed laboratory population kept in the lab for at least ten generations (*Astbur* and *AstburLAB*, respectively). According to stable isotopes and gut content analysis, *A*. *burtoni* is omnivorous, but largely feeds on algae and plants [36]. Lab diet consisted of flake food twice a day and frozen artemia once a day.

For most species we individually profiled the gut microbiota of five individuals, with the exception of *Haptri* (four individuals) and *Perecc* (one individual) (S1 Table). All wild conspecifics were captured in the same locality, except for *Plestr*, whose specimens came from three distinct sites (geographically nearby). Specimens were trapped in nets and processed within an hour from catch. Full fish specimens were preserved in ethanol 100%, with their ventral side cut-opened for facilitating ethanol flow to internal organs. All sampling procedures in Lake Tanganyika and experimental manipulations *in situ* followed strict ethical guidelines and were approved as part of obtaining the field permits issued by the Lake Tanganyika Research Unit, Department of Fisheries, Mpulungu, Republic of Zambia, taking into account the 3Rs strategy (Reduction, Replacement, Refinement).

Specimens of *AstburLAB* came from an individual tank with standardized conditions of constant water temperature of 26°C, pH 7, and a 12:12 h light:dark cycle. Specimens were euthanized with MS 222 using approved procedures (permit nr. 2317, issued by the cantonal veterinary office from Switzerland) and directly processed for DNA extractions.

It is worth mentioning that in all cases we specifically sampled only adult individuals; because the dynamics of the cichlid gut microbiota through life stages are unknown, we assume their gut microbiota to be relatively stable at adult stage, according to study in humans [44].

All experiments were approved by the cantonal veterinary office from Switzerland and by the University of Basel veterinary office (permit nr. 1010H). The institution has been approved as research unit according to EU guideline 92/65/EWG (nr. CH-I-BS017) and CITES (nr. CH018).

### DNA extractions and 16S rRNA pyrosequencing

All cichlid species in this study are relatively small allowing sampling and easy processing of the whole guts. The entire digestive tracts, including stomachs, were dissected: stomach content was carefully flushed out with sterile water, while intestine content was left untouched. This procedure maximizes the representation of gut microbes, including those from the epithelial mucus layer, despite putatively including some transient bacteria through ingesta.

DNA was extracted from individual guts using a modified version of a repeated beat-beating plus column (RBB+C) protocol [45]. Specifically, gut tissues were transferred to 800 μl lysis buffer with 0.3 gr. of 0.1 mm zirconia-silica beads, then homogenized with a FastaPrep bead beater (MP Biomedicals) at 5.0 M/S, 2x 45 sec and incubated at 70°C for 20 min. Samples were then centrifuged at 14,000 rpm, and supernatant stored separately. After addition of 400 μl lysis buffer, pellet was bead-beaded once more for 45 sec and supernatants were combined. Approximately 20–25 μl of proteinase K (to a final of 10 mg/mL) were added to the supernatant followed by incubation at 55°C for 45 min. Residues were eliminated with ammonium acetate (to a final of 2.5 M) and DNA precipitated with isopropanol and standard ethanol washes. DNA was diluted in TE (pH 7.0) and cleaned with Qiagen columns (DNeasy Blood and Tissue kit).

Species taxonomic classification was confirmed by amplification of the mitochondrial gene D-loop with primers L-PRO-F/TDK-D and sequence comparison against published sequences in Genbank.

Two 16S rRNA fragments were sequenced with the titanium LIB-L kit for unidirectional pyrosequencing (454/Roche) using slightly modified standard primer pairs 8F/519R and 356F/1064R, which amplify regions V1–V3 and V3–V5, respectively (S2 Table). To avoid hairpins formation between primers and barcodes, all barcodes-forward primers combinations were previously checked for intra-folding structures using mFold application (http://mfold.rna.albany.edu/?q=mfold) and the 10 best combinations were chosen. PCR was performed on a 20 μl-volume using 1X of Qiagen Multiplex PCR kit, including HotStarTaq DNA Polymerase, 0.2 μM of primer and 1 μl of DNA (around 100–150 ng/ μL). Conditions were set to 15 min at 95°C, followed by 32 cycles of 45 sec at 94°C, 45 sec at 58°C and 1 min 30 sec at 72°C, plus final extension of 10 min at 72°C. Each sample was amplified in three replicates, plus a negative control (water only) and amplicons combined. Amplicon size and concentration were checked on Bioanalyzer (2100 Agilent Technologies).

For each 16S amplicon, specimens were individually barcoded and pooled at equimolar concentration in three 454 libraries (ten barcodes each library) after purification with AMPure beads (Agencourt Beckman Coulter) (S2 Table). Conspecifics were partitioned across libraries to minimize methodological biases. Libraries were sequenced on a Genome Sequencer FLX system at the Norwegian Sequencing Center (NSC).

### Sequence analyses

Reads were inspected using the online tool PRINSEQ v0.20.4 and trimmed with the sfffile tool (454-Roche) according to the quality graphs. After trimming, the two 16S fragments were approximately 320 bp and 470 bp long and fully encompassed V1-V2 and V3-V4, respectively (hereafter named V12 and V34). The six libraries were then similarly processed through QIIME v1.7.0 [46]. Sequences were filtered and demultiplexed using Split_libraries (settings-M3-b10-reverse_primer_mismatches 3-w50-g-s20) followed by denoising with denoise_wrapper.py. Each library was inflated and checked for chimeras using UCHIME v4.2, implemented in Usearch, against the ChimeraSlayer reference database (“gold”) in the Broad Microbiome Utilities. Chimeras were discarded. All same-amplicon sequences were combined and de-novo assigned to operational taxonomic units (OTUs) based on 97% identity default threshold using uclust [47] and Trie prefilter. A representative sequence for each OTU was picked (i.e. most abundant) and assigned to taxonomy based on the Greengenes database gg_13_5_otus, retraining with the RDP classifier (0.8 confidence level). We retained only OTUs shared across two or more samples, plus all singletons represented by >3 reads. All Cyanobacteria, except members of the new lineage of Melainabacteria (see Results), were removed from the dataset as putatively derived from ingested material. We cannot, however, fully exclude sampling of environmental bacteria, given the inclusion of digesting matter. While this is a common issue when studying wild samples, we also note that the current classification of bacteria as either gut-associated or free-living remains a challenging task and is currently being revised (e.g. for Planctomycetes [48] and Melanobacteria [49]).

Sequences were aligned with PyNAST default parameters [46] using the reference database “core_set_aligned.fasta” from Greengenes website and default lanemask. Alignments were further trimmed and a phylogenetic tree was built with FastTree [50].

The three alpha diversity indexes, Chao1, Shannon index, and Phylogenetic diversity (PD) Whole Tree metrics, were estimated on rarefied OTU tables at 500 reads/sample to a sub-sampling depth determined as the minimum number of sequences per sample, and 10 iterations. Beta diversity (Unifrac and binary_Jaccard) was measured using the script jackknifed_beta_diversity.py on an even-depth rarefaction for all samples determined as the minimum number of sequences in each library and visualized through principal coordinates analysis (PCoA). We used the script dissimilarity_mtx_stats.py to calculate means and standard deviations for all the rarefied unweighted unifrac distance matrices. With the script make_distance_boxplots.py we generated distance boxplots for comparisons among all host species and performed two-sample t-tests (with Bonferroni correction for multiple comparisons) for all pairs of boxplots to help determine which boxplots (distributions) were significantly different.

Procrustes analyses were performed to compare beta diversity patterns of UniFrac distances between V12 and V34, with Monte Carlo simulations (1000 permutations).

Rarefaction curves on the observed number of OTUs were calculated on a sub-sampling of 500 reads/sample to a sub-sampling depth determined as the minimum number of sequences in each library and 10 iterations. Graphs were plotted with function ggplot in R.

The core microbial taxa and OTUs were computed for each species as well as for all cichlids using an in-house R script. The core was defined as the microbial component shared by ≥80% of the specimens, at least one individual per species and, in case of core taxa (and not OTUs), consistently recovered by both libraries to minimize potential errors in taxonomic assignment of the two 16S fragments.

### Statistical analyses

Alpha diversity estimates were compared among host species through a Mann-Whitney U-test based on average species values obtained by averaging specimen values after rarefaction.

To test whether the species relative core length (i.e. percentage of core OTUs shared by at least 80% of conspecifics over the total number of OTUs per fish species) was larger than expected by chance, we applied a permutational test. Observed relative core lengths were compared with those resulting from shuffling species labels and calculating relative core length on five randomly chosen individuals (1000 times).

Multivariate analyses were used to explore whether microbiota composition at distinct taxonomic levels (OTUs, species, genus, family, order and phylum) was significantly different among fish species, tribes and diet.

Multivariate data were visualized using PCoA with cmdscale function of stats R package, while differences among groups were tested using distance-based permutational MANOVA with adonis function of vegan R package. First, a PCoA and multivariate test was performed on the entire OTU database using presence-absence data and the Jaccard distance, and these results were compared to PCoAs based on weighted and unweighted unifrac distances. Secondly, analyses were performed on relative abundance data for different taxonomic levels using the Manhattan distance on arcsin transformed relative abundance data. The arcsin transformation is often used to normalize fractional data and gives more weight to both the lowest and highest values. The Manhattan distance is equivalent to the Bray-Curtis distance applied to fractional data, which is widely applied in community ecology.

The indicator value of bacterial taxa with respect to fish species, genus or tribe was calculated as per Dufrene and Legendre (1997) as the product of the relative frequency and relative average abundance in clusters (i.e., host species, genus or tribe). In general terms, a bacterial taxon gets a high indicator value if it tends to be present only in one cluster but not in others, and it is shared by most members of that cluster. Indicator values were calculated using function indval of labdsv R package. Concordance between libraries was set as a filter for retaining taxa in order to exclude potential methodological biases.

## Results

### Biodiversity of the cichlid gut microbiota

We characterized the gut microbiota of a total of six cichlid species from wild populations of lake Tanganyika and a surrounding river (S1 Table): five species belonging to the tribe Perissodini (*Hapmic*, *Haptri*, *Plestr*, *Permic* and *Perecc*) and one species to the distant tribe Tropheini (*Astbur*), including a captive strain of the same species (*AstburLAB*).

After quality filtering, we obtained around 240,000–250,000 reads per each 16S fragment and an approximate mean of 40,000 high quality reads per species/library (S3 Table). After singletons removal, V12 identified 970 OTUs, which were assigned to 25 phyla, 48 classes, 83 orders, 123 families and 121 genera. V34 identified 763 OTUs, which were assigned to 22 phyla, 49 classes, 85 orders, 134 families, and 136 genera. Overall, the two libraries provided a similar spectrum of taxonomic diversity, with V34 recovering less OTUs but more families and genera.

Number of OTUs varied substantially among conspecifics and between libraries, although this variation was not correlated to the sequencing effort. Nonetheless, total number of unique OTUs per species was comparable between libraries and ranked similarly (except for *Permic*), with *Astbur* being the most OTU-rich (approximately 700 OTUs). According to rarefaction curves on the number of observed OTUs, V12 and V34 recovered a highly similar pattern of between-conspecifics and among-species diversity (S2 Fig). We clearly undersampled the biodiversity of *Astbur*, while the sampling depth was likely sufficient to characterize the microbiota of most Perissodini specimens.

**Fig 1:**
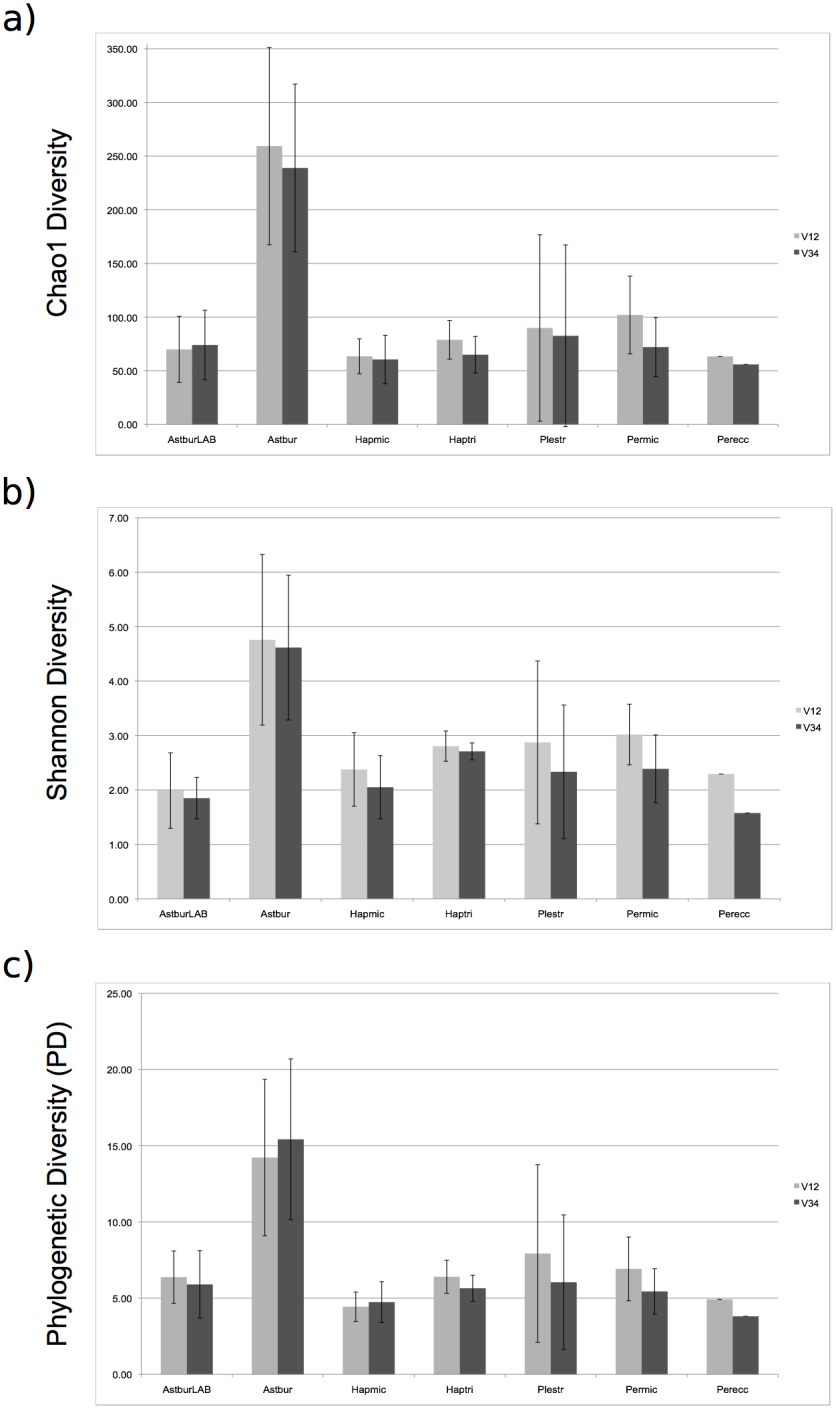
Mean alpha diversity estimates per species (Chao1 (a), Shannon (b) and PD whole metric (c)), and standard deviation across conspecifics (bars). The two libraries significantly correlated in the pattern of diversity across-species for all three alpha indexes. *Astbur* carried the most biodiverse microbiota, significantly distinct from all other species and the same species kept in laboratory (i.e. *AstburLAB*) (p-value<0.05, all indexes, both libraries). Differences among Perissodini species were not statistically relevant (p-value>0.05, all indexes).

In terms of alpha diversity, the two 16S fragments provided highly similar estimates per species (Fig 1) and the same pattern of diversity among-species for all three alpha indexes (correlation analysis with linear regression, R² = 0.97037 for Chao1, 0.9087 for Shannon and 0.95627 for PD). Among all samples, *Astbur* carried the richest and most phylogenetically diverse microbiota of all species (significantly distinct from all members of the tribe Perissodini, Mann-Whitney U-test, p-value<0.05 for both V12 and V34, all three diversity indexes). Notably, the same species kept in laboratory conditions (*AstburLAB*) displayed a highly reduced microbiota biodiversity with respect to the wild population (above 50% in all indexes, p-value<0.05). Within the tribe Perissodini (*Hapmic*, *Haptri*, *Perecc*, *Plestr* and *Permic*) microbial diversity among species was not statistically different (all pairwises, p-value>0.05) (Fig 1).

In what follows, we first explore the shared microbial components among cichlids (i.e. core) at family (Cichlidae) and species level, with the goal of detecting signature of the host phylogenetic relatedness. We subsequently examine their distinctive features (qualitative and quantitative), putatively associated to variation in trophic niches. In both cases we use concordance between the two 16S libraries to exclude methodological biases.

### Cichlid core taxa

**Fig 2:**
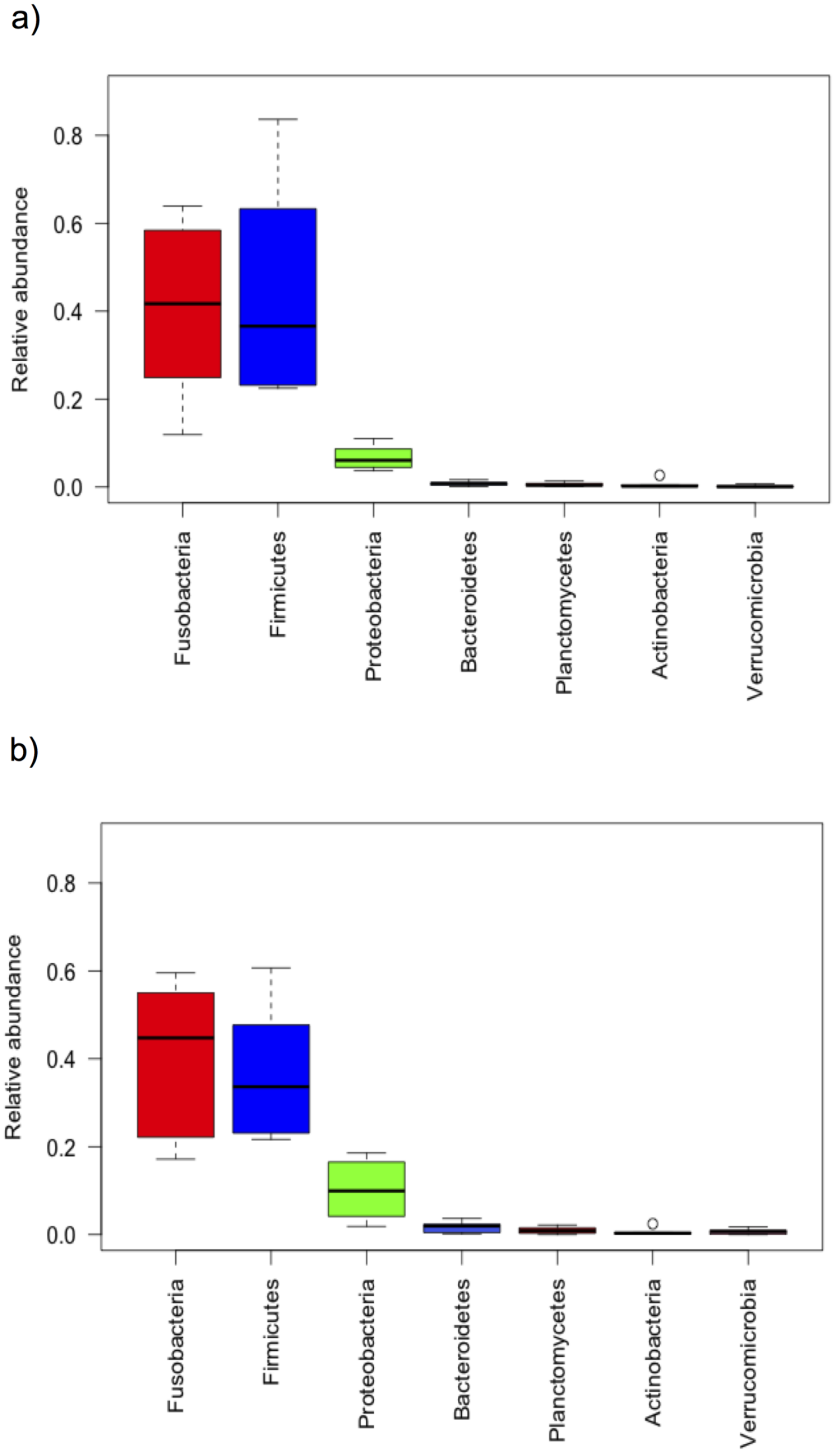
Relative abundance of the seven cichlid core phyla in V12 (a) and V34 (b). Interquartile ranges (25th and 75th percentiles) and whiskers show data dispersion across species averages. Medians are shown as central horizontal lines. The two libraries returned a highly concordant pattern of core phyla abundance: the cichlid gut microbiota is dominated by Fusobacteria, Firmicutes and Proteobacteria, with the first two phyla largely fluctuating in relative abundance across species. Bacteroidetes, Planctomycetes, Actinobacteria and Verrucomicrobia are consistently less represented in all species (overall contributing less than 1% of the total reads).

Seven phyla constituted the cichlid core (Table 1): Firmicutes, Fusobacteria, Proteobacteria, Bacteroidetes, Actinobacteria, Planctomycetes, and Verrucomicrobia, together contributing more than 90% of the total reads per fish species (Fig 2). Fusobacteria and Firmicutes represented the dominant component in both libraries (with a pooled median of 78% of reads per species, each library), while largely fluctuating in relative abundance across species. Proteobacteria were consistently less represented, but slightly more abundant in V34 (median 6.1% for V12 and 9.9% for V34). The remaining four phyla occurred at remarkably lower abundance (<1%), although consistently in all species.

**Table 1:** Cichlid core bacterial taxa, defined by presence in at least 80% of the individuals (i.e. 20/25, excluding *AstburLAB*), a minimum of one representative per species and consistently in both 16S libraries.

Phylum	Class	Order	Family	Genus	Species
Actinobacteria	Actinobacteria	Actinomycetales	Aeromonadaceae	Cetobacterium	*Cetobacterium somerae*
Bacteroidetes	Alphaproteobacteria	Bacillales	Clostridiaceae	Clostridium	*Clostridium perfringens*
Firmicutes	Bacilli	Bacteroidales	Enterobacteriaceae	Plesiomonas	*Plesiomonas shigelloides*
Fusobacteria	Bacteroidia	Burkholderiales	Fusobacteriaceae	Turicibacter	
Planctomycetes	Betaproteobacteria	Clostridiales	Lachnospiraceae		
Proteobacteria	Clostridia	Fusobacteriales	Neisseriaceae		
Verrucomicrobia	Fusobacteria	Turicibacterales	Pirellulaceae		
	Gammaproteobacteria		Rhodobacteraceae		
	Planctomycetia		Turicibacteraceae		

Further down in the classification, the cichlid core comprises nine bacterial classes, seven orders, nine families, and four genera (Table 1), most of which are common associates of the vertebrate gut. Three core species were detected: *Cetobacterium somerae*, *Clostridium perfringens*, and *Plesiomonas shigelloides*, which also represented the three overall most abundant species in both 16S libraries. Few core OTUs per bacterial species were responsible of this high abundance; *C*. *somerae* single OTU, in particular, represented more than 30% of all reads in both libraries (OTU-1052 for V12 and OTU-370 for V34, with the same best BLASTN hit to a fish gut microbe, S4 Table) and was by far the most abundant sequence in all individual fish species and most specimens, including the domesticated one. While suggestive of an amplification bias, the consistent recovery of this OTU from multiple DNA samples and both amplicons indicate it is rather a real quantitative trait.

In terms of core OTUs, wild cichlids (80% of all individuals, at least one individual per species) shared 14 OTUs in each library (Table 2, for details see S4 Table). Of these, nine OTUs were putatively environmental, according to best BLASTN matches in the nt database; nonetheless, seven of them were also detected in the laboratory species *AstburLAB* suggesting that their source might not be environmental. The remaining core OTUs (19) typically gave best matches to gut microbes of other fishes or higher vertebrates (identity ≥ 98%).

**Table 2:** Cichlid core OTUs (80%).

OTU ID^1^	Taxon^2^	Best hits (≥ 98% identity), AccNo	Source
82, 1052; 370	*Cetobacterium somerae*	HG326498, KF256025; KC601503	fish gut, rodent gut
; 89, 513, 580	*Clostridium perfringens*	GU293218, EU181010	fish gut, seagull feces
1599; 137	*Plesiomonas shigelloides*	JX512414; JN033047	fish gut; fish gut
558; 1144	*Turicibacter sp*.	HM630213; AJ852321	fish gut; insect gut
179, 1688, 255, 1373;	*Clostridium XI* sp.	JF573566, JF656416, EU475605	rumen fluid, babirusa feces
; 701	*Aeromonas* sp.	JX860615	fish gut
410; 831	Neisseriaceae	KC601171; DQ817700	fish gut
57; 1270	Lachnospiraceae	HM630161	fish gut
730; 416	Clostridiales	JN397966	Environmental
927; 1478	Clostridiaceae	KC324979; JN397963	Environmental
1808	Gemmataceae	KF927577	Environmental
1262	*Achromobacter* sp.	JF397248	Environmental
1405	*Bacillus* sp.	AB968095	Environmental
154, 478	Pirellulaceae	AB930498; JQ958724	Environmental

^1^ OTUs from V12 (before semicolon), and OTUs from V34 (after semicolon). In case of OTUs returning the same best hit (putatively belonging to the same bacterial strain), only one AccNo is indicated.

^2^ Correct classification was verified by concordance of sequence matches to several databases (RDPII, nt and Greengenes).

Considering only the core OTUs with matches to host-associated microbiotas, altogether cichlids shared at least eight core species: *C*. *somerae*, *C*. *perfringens*, *P*. *shigelloides* and five or more unclassified species of the genera *Turicibacter*, *Clostridium* XI (Firmicutes) and *Aeromonas* (Proteobacteria), and of the families Neisseriaceae (Proteobacteria) and Lachnospiraceae (Firmicutes) (Table 2).

Remarkably, the laboratory strain *AstburLAB* harboured the same core taxa found for wild cichlids (except for the phylum Verrucomicrobia and the family Enterobacteriaceae, Table 1) and all host-associated core OTUs shown in Table 2.

### Intra- and interspecific OTU core size

**Fig 3:**
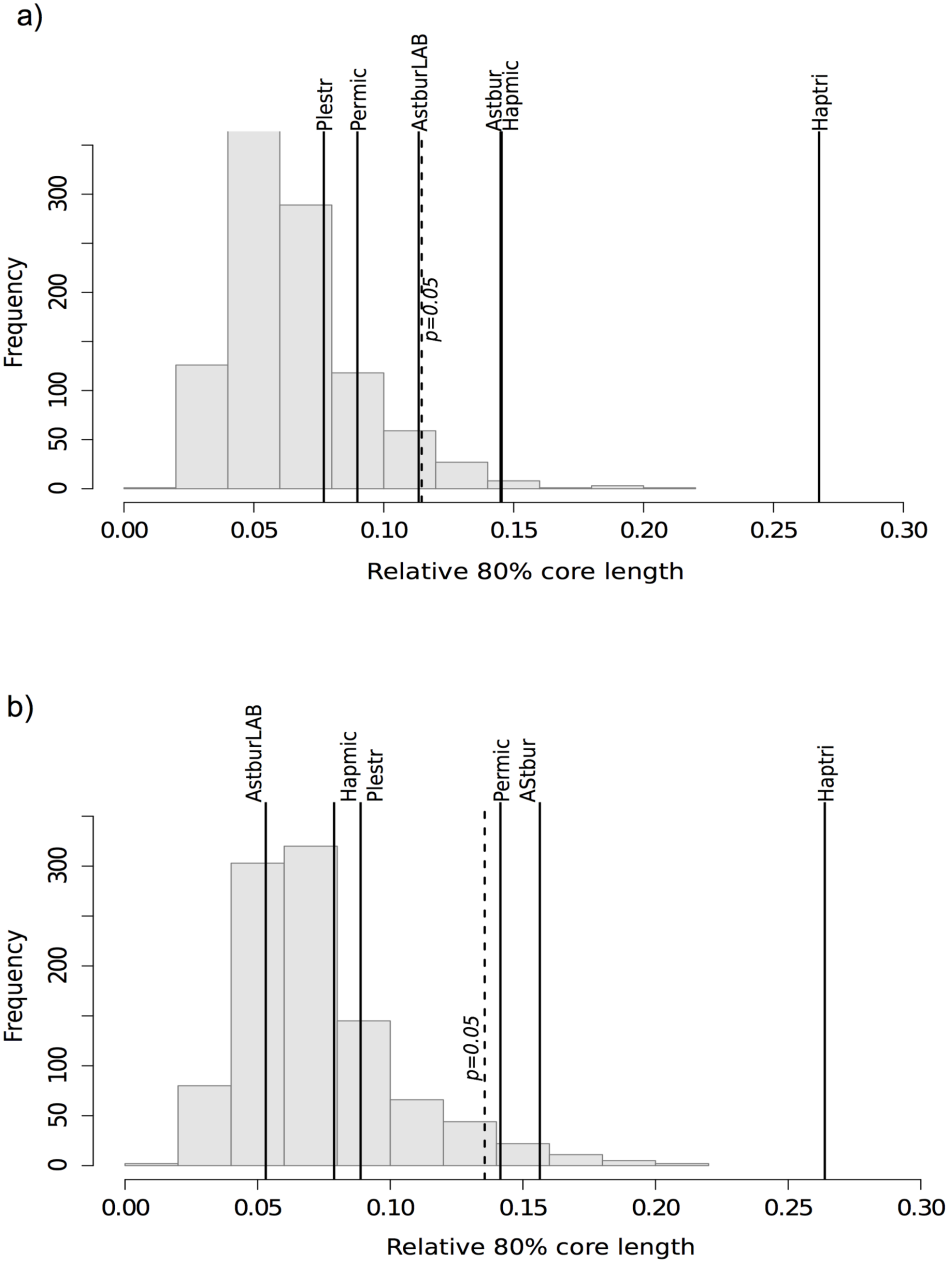
Observed intraspecific core length (i.e. relative proportion of shared OTUs across 80% of conspecifics, shown as vertical lines), compared to random sampling of the whole dataset (barplots, 1000 permutations, p<0.05) for V12 (a) and V34 (B). There is a general trend for increase relative core length among conspecifics compared to random individuals, with few species carrying a significantly larger core in one or both libraries (*Astbur*, *Haptri*, *Hapmic* and *Permic*, p<0.05). *Perecc* was excluded from the analysis because it contributed with only one individual.

Conspecifics shared between 5.5% and 29.6% of their total OTUs, with an average of 13–15% (for presence in 80% of the specimens). Is this fraction significantly larger than what we would expect based on a random sampling of individuals? Is it larger in intraspecific *versus* interspecific comparisons? Based on a permutational test, the intraspecific core microbiota was significantly larger than expected by chance for some species (*Hapmic*, *Haptri* and *Astbur* for V12, Fig 3a; *Haptri*, *Astbur* and *Permic* for V34, Fig 3b; 1000 permutations, p<0.05,) or typically larger than the mode (although not statistically significant) for the others, suggesting a trend of nonrandom OTUs transfer at intraspecific level. Moreover, the proportion of shared OTUs was overall larger in intraspecific than interspecific pairwises (Wilcoxon test with continuity correction W = 7859, p-value = 9.73e-05 for V12; W = 7016, p-value = 0.01506 for V34) indicating that such proportion decreases with increased host phylogenetic distance. This pattern was partly biased by the interspecific pairwise comparisons between the two distant tribes; however, when only Perissodini were analyzed, differences in core size between intra and interspecific comparisons remained partly significant (W = 3464.5, p-value = 0.009889 for V12; W = 3150, p-value = 0.1018, not significant for V34).

Although the core OTU size appears to be larger in conspecifics compared to random pools of individuals and to interspecific comparisons, its taxonomic composition is not strictly host-specific. Indeed, intersection of core OTUs from distinct cichlid species shows large microbial promiscuity, with Perissodini altogether sharing over half of their individual species core OTUs.

### Beta-diversity clustering of the cichlid gut microbiota

**Fig 4:**
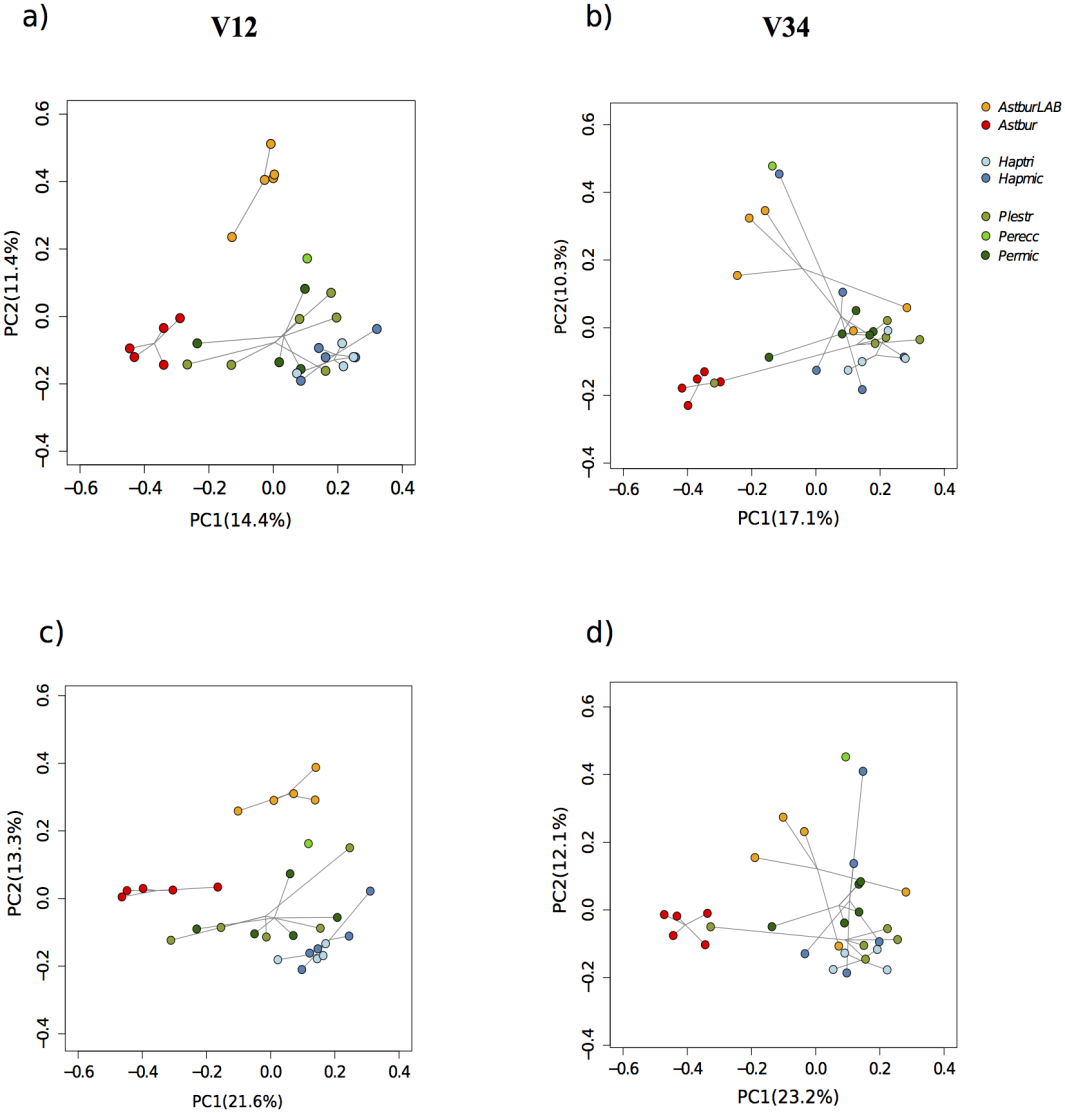
Principal coordinate analysis of beta diversity estimates for whole microbial communities of the 30 specimens analyzed (individual circles) based on binary Jaccard (a, b) and unweighted unifrac distances (c, d) for V12 (a, c) and V34 (b, d). Lines connect circles with the species centroid; species are colour-coded (see Legend). Despite few discrepancies between libraries, all plots illustrate two main clusters: the member of the tribe Tropheini, *Astbur* (in red), and the Perissodini species (*Haptri*, *Hapmic*, *Permic*, *Perecc* and *Plestr*), which largely superimpose in their microbiota space. The laboratory species *AstburLAB* carries a remarkably distinct microbiota from its wild conspecifics (*Astbur*).

The two 16S fragments returned comparable microbial community structures according to Procrustes analyses based on both unweighted and weighted PcoAs (p = 0.0001 for both analyses, M^2 = 0.516 and M^2 = 0.484, respectively). Overall, the cichlid gut microbiota clustering largely resolved the main host subdivision between the member of the tribe Tropheini (*Astbur*) and all members of the tribe Perissodini (Fig 4 and S3 Fig). This clustering was quite robust and recovered with both OTU similarity (binary Jaccard, permutational Anova p<0.01 both libraries, Fig 4a and 4b) and phylogenetic-based approach (Unweighted Unifrac, multiple Student’s two-sample t-tests with Bonferroni correction, p<0.05 based on V12, Fig 4c). The microbiota composition of *Astbur* significantly differed also from that of the same species raised under laboratory conditions; notably, the intraspecific mean distance between the *AstburLAB* and *Astbur* was higher than the mean distance within the entire Perissodini tribe, indicating a dramatic effect of captivity on the microbiota composition.

A few discrepancies between libraries can be observed, mainly in the placement of *AstburLAB* specimens that clustered separately from all other species in V12 (Fig 4a and 4c) but partly overlapped with the Perissodini species in V34 (Fig 4b and 4d), although they remained consistently separated from the wild specimens of the same species.

Interestingly, the Perissodini species showed a large overlap of their microbiota profiles, despite important differences in diet habits: the unweighted unifrac tree showed that, except for *Haptri*, the microbial communities of conspecifics did not cluster together (S3 Fig) and that the intraspecific Unifrac distances were not significantly distinct from interspecific distances for any Perissodini species pairwise comparison (two-sided Student's two-sample t-test p-value >0.05 with Bonferroni correction, both libraries).

### Multivariate analyses, indicator taxa and OTUs for discriminating among gut microbiotas

All microbial components were dissected to explore the single contribution, in terms of relative proportions (including presence/absence), of each bacterial taxonomic “category” (i.e. phylum down to genus) to the host taxonomic subdivision (in tribe, genus and species) and diet (scale *versus* zooplankton feeders). According to multivariate analyses, all bacterial levels were able to significantly resolve the main subdivision between Perissodini and *Astbur*, as well as between *Astbur* and *AstburLAB* (permutational Anova, p<0.0001, both libraries), with “Phylum” being less powerful in this respect (p<0.01). “Bacterial genus” overall represented the best indicator for microbiota resolution at host species level, with most cichlid species being significantly discriminated (p<0.01). At upper taxonomic levels (from order to Phylum) the separation among Perissodini species became progressively unresolved. No bacterial taxonomic level was differentially enriched between the two diet classes.

We also identified those specific bacterial taxa and OTUs that showed a significant enrichment (i.e. indicator taxa and OTUs) with respect to each level of the host taxonomy and between diets. Based on the complete list of indicator taxa (p<0.05, S5 Table), *Astbur* carried the most peculiar microbiota: more than 85% of all the indicator taxa were assigned to this species and a large proportion of them was exclusive. For simplicity, we restricted the analyses to the list of the ten best indicator values per taxonomic category for *Astbur*, while including all significant indicator taxa for the remaining species (p<0.05) (Table 3). Two phyla were a signature of the *Astbur* microbiota: Chloroflexi (and the associated genus Caldilineaceae), and the candidate subdivision TM7, which were virtually absent in Perissodini as well as in *AstburLAB*. The *Astbur* gut microbiota was also particularly enriched in Lactobacillales and its major families, Leuconostocaceae, Streptococcaceae and Lactobacillaceae, when compared to either single species or the whole Perissodini tribe. Other significantly enriched taxa were the family methylocystaceae (Rhizobiales, and associated genus *Methylosinus*), and the genus *Demequina* (family Cellulomonadaceae). A most distinctive feature of *Astbur* was the exclusive presence of Cyanobacteria from the order YS2, even though they occurred at low abundance in the microbiota (165 reads). Notably, all 16 OTUs (from V12 and V34 altogether) associated to this order clustered with the recently proposed novel group of gut Melainabacteria [49] (S4 Fig).

**Table 3:** Summary of indicator taxa per taxonomic category in comparisons (a) across host species, (b) host genera (Perissodini only) and (c) between tribes (p<0.05 and indicator value ≥0.6 for at least one library).

			Indicator Value^3^	
	taxon	cluster^1^ ^,^ ^2^	V12	V34
**a) Across species**				
**Phylum**	TM7	*Astbur*	0.9884	0.8646
**Order**	Lactobacillales	*Astbur*	0.9469	0.9619
	Aeromonadales	*Haptri*	0.7167	0.7062
	Methylacidiphilales	*Haptri*	0.9051	0.7972
**Family**	Methylocystaceae	*Astbur*	0.8831	0.8533
	Streptococcaceae	*Astbur*	0.9752	0.9808
	Aeromonadaceae	*Haptri*	0.7354	0.003
	LD19	*Haptri*	0.9051	0.034
**Genus**	Demequina	*Astbur*	0.7551	0.8
	Lactococcus	*Astbur*	0.7656	0.9821
	Methylosinus	*Astbur*	0.8896	0.8544
	Propionicimonas	*Astbur*	0.966	0.7414
	Deefgea	*Haptri*	0.8604	0.8787
**b) Across genera**				
**Family**	Clostridiaceae	*Perissodus*	0.6064	0.4814
**Genus**	Clostridium	*Perissodus*	0.7411	0.527
**c) Between tribes**				
**Phylum**	TM7	Tropheini	0.9971	0.9827
	Chloroflexi	Tropheini	0.9635	0.9945
**Order**	YS2	Tropheini	1	0.7732
	Lactobacillales	Tropheini	0.9987	0.998
	Chromatiales	Tropheini	0.995	0.986
	Caldilineales	Tropheini	0.9642	0.9989
	Rhodobacterales	Tropheini	0.9099	0.9023
	Rhizobiales	Tropheini	0.9072	0.9414
	Solirubrobacterales	Tropheini	0.8933	0.8954
	Methylococcales	Tropheini	0.8	0.8748
	Gaiellales	Tropheini	0.7636	0.971
	Enterobacteriales	Perissodini	1	0.9839
**Family**	Lactobacillaceae	Tropheini	1	0.7996
	Leuconostocaceae	Tropheini	0.9996	0.7996
	Streptococcaceae	Tropheini	0.9985	0.9978
	Chromatiaceae	Tropheini	0.995	0.986
	Methylocystaceae	Tropheini	0.9702	0.9645
	Caldilineaceae	Tropheini	0.9642	0.9989
	Isosphaeraceae	Tropheini	0.9575	0.8575
	Rhodobacteraceae	Tropheini	0.9099	0.9023
	Hyphomicrobiaceae	Tropheini	0.8793	0.9246
	Enterobacteriaceae	Perissodini	1	0.9839
	Shewanellaceae	Perissodini	0.65	0.65
**Genus**	Caldilinea	Tropheini	0.976	0.9988
	Methylosinus	Tropheini	0.9713	0.9649
	Rhodobacter	Tropheini	0.9359	0.9353
	Hyphomicrobium	Tropheini	0.8759	0.9532
	Lactobacillus	Tropheini	1	0.7996
	Propionicimonas	Tropheini	1	0.799
	Leuconostoc	Tropheini	0.9993	0.7996
	Lactococcus	Tropheini	0.7982	0.9978
	Luteolibacter	Tropheini	0.7769	0.9104
	Plesiomonas	Perissodini	1	0.9835
	Shewanella	Perissodini	0.65	0.65

^1^ Host taxon with significant bacterial enrichment.

^2^ For clusters "*Astbur*" and "Tropheini" only the ten best indicator taxa per taxonomic category are listed, chosen as those with the highest sum of values from both libraries and p<0.01. A complete list is provided in S5 Table.

^3^ An indicator of value 1 indicates that a bacterial taxon is exclusive of one cluster and shared by all members of that cluster.

Perissodini only distinguished from the Tropheini species by a significant enrichment in *Plesiomonas* (Enterobacteriales, p<0.01) and *Shewanella* (p<0.05) (Table 3).

In the across-species comparison within Perissodini, only *Haptri* featured significant taxa enrichment for the families Aeromonadaceae and LD19 and the genus *Deefgea* (family Neisseriaceae). Besides these exceptions, individual Perissodini species do not display distinctive features that were significantly recovered by both 16S libraries.

The list of indicator OTUs reflects a similar scenario. Again, in the across-species comparison the majority of differentially represented OTUs were found in *Astbur* (>80%, S6 Table). A proportion of these indicator OTUs were exclusive or nearly unique of this species (indicator value approximate or equal to 1), signifying that *Astbur* microbiota differed, at least in part, by presence of additional OTUs rather than by a differential abundance of shared OTUs across cichlids. Within Perissodini, among-species discrimination is only seen in the enrichment of few OTUs: according to both libraries, *Haptri* showed a higher occurrence of one Defgea and one Aeromonadaceae OTU (also recovered by the indicator taxa, S5 Table), while *Permic* was significantly enriched in *C*. *perfringens* (OTU-89), although based only on V34 (S6 Table). Beside these few OTUs, Perissodini overall did not significantly differ in either their quantitative or qualitative OTU representation, reflecting the findings from the indicator taxa and PcoA analyses based on the whole microbiota dataset (S5 Table and Fig 4).

When a comparison is done at tribe level, more than 95% of the indicator values were assigned to *Astbur* (both libraries), and 50 to 65% of them were nearly exclusive of this species (S7 Table). According to both libraries, Perissodini largely distinguished from the Tropheini species only for enrichment in *P*. *shigelloides* and *C*. *perfringens* OTUs.

### Indicator values for diet discrimination

Strikingly, we did not detect any indicator taxa (at any level of bacterial taxonomy) that were exclusively found or enriched in either zooplankton or scale-eaters according to both libraries (p<0.05). A significant difference emerged only in contrasts across Perissodini genera: the two scale-eaters of the genus *Perissodus* (*Permic* and *Perecc*) displayed a significant enrichment in the family Clostridiaceae and mainly in the genus *Clostridium* when compared to all other genera (p<0.05, Table 3). The OTU that most contributed to this pattern, according to V12, was OTU-1421, classified as *C*. *perfringens* (value 0.904, p = 0.011). Two other OTUs were differentially represented between the two diet categories and specifically enriched in zooplankton feeders, again according only to V12: OTU-1599 (P. *shigelloides*, value = 0.895, p = 0.001) and OTU-1516 (Lachnospiraceae member, value = 0.949, p = 0.02).

## Discussion

### Dynamics of the gut microbiota during dietary transition

The primary pattern in the cichlid microbiota community clustering followed the host taxonomic subdivision between the two tribes, Perissodini and Tropheini, in contrast with the larger diversity of diet habits of the sampled species. Despite substantial intraspecific variation, at species level the Perissodini gut microbiota largely superimposed in their phylogenetic profiles: none of the five species significantly differed from all other members of the tribe in terms of microbial alpha and beta diversities (Figs 1 and 4) or by presence of unique taxa and OTUs (Table 3 and S5 Table).

The few gut microbiota differences detected within this tribe were quantitative. Specifically, the Perissodini species *Haptri* showed a couple of significantly enriched taxa and OTUs when compared to the other members of the tribe. Furthermore, we observed an increased representation of Clostridiaceae (and mainly of few *Clostridium* OTUs) in the true scale-eaters *Permic* and *Perecc* when compared to the zooplankton feeders *Haplotaxodon*. This overall suggests that simple changes in taxa relative abundance, rather than acquisition of novel bacteria taxa, might account for the microbiota compositional transition among Perissodini species. Nevertheless, considering the conservative filtering applied for retaining an indicator taxon (i.e. recovering by both 16S libraries), it is possible that a differential primers bias for taxa amplification might be in part responsible for this reduced overlap between datasets and therefore we cannot exclude that, with an increase in sequencing effort, other minor qualitative and quantitative differences among species might emerge.

To date, the studies of microbiota dynamics following speciation and diet shifts in wild vertebrates have been limited [10, 18, 19], and virtually absent in fishes [22, 23], precluding fine comparative analysis at this stage. A recent study on surgeonfishes represents a notable exception [23] and indicates diet as a major player in shaping the fish gut microbial communities (although phylogeny also contributes in part to the observed clustering). This study also points to the need for a better characterization of the diet niches and ecology for individual species and populations in the wild, a field that is still poorly investigated. We also urge the use of a standardized method for gut microbiota sampling, besides a unified protocol for sequencing. The Authors of this study indeed only sampled gut content, likely missing part of the critical microbial community present in the epithelial mucus layer and potentially overappresenting the transient bacteria in the ingested food (hence, a major role of diet in shaping the microbiota). Recent studies in sticklebacks, sampling all gut tissues and content, are indeed clearly showing that fish gut microbiota does not simply reflect the microbial composition of the consumed food and other factors, such as the host genetics, might play a role [12, 25]. In our study, the similarity of gut microbiota taxonomic composition seen among Perissodini species, despite diet differences, is concordant with these recent findings.

At functional level, the apparent correlation between the high abundance of *Clostridium* and strict lepidophagy might be attributed to scale metabolism. Collagen, the main component of ctenoid scales consumed by scale-eaters, is typically broken down by collagenases found in the stomach [51], but some bacteria of the genus *Clostridium* also possess different types of this enzyme [52], thus putatively relating the high abundance of *Clostridium* found in scale-eaters to an increasing demand for collagen degradation. As morphology of the cichlid gut is not well known at this stage, we cannot anticipate whether most of the collagen hydrolysis occurs before reaching the intestine (i.e. in the stomachs, by endogenous enzymes) or in the intestine. Unknown is also the relative contribution of microbial *versus* host digestion. Scale-eaters typically present shorter guts than zooplankton-feeders, only suggesting a faster transit in the intestine [37]. Clearly, functional studies are needed to properly address this point. Lepidophagy has independently evolved in the other two African great lakes, Malawi and Victoria [39], and future characterization of the gut microbiota of these unrelated species will shed light on the microbial adaptation to this diet.

Interestingly, the Perissodini microbiota was essentially represented within that of the Tropheini species *A*. *burtoni*, as revealed by the virtual lack of indicator taxa and OTUs exclusive of the Perissodini tribe when compared to this outgroup species. *A*. *burtoni* is omnivorous, while mostly feeding on plants and algae [36], and it alone exhibits most of the gut microbial biodiversity seen in cichlids (S2 Fig), altogether with several nearly exclusive bacterial taxa (such as Lactobacillales, Rizhobiales and gut Melainobacteria). It has been postulated that increasing herbivory can enhance microbial biodiversity, as documented in notothenioid fishes [33] and mammals in general [8, 10]. Cichlids might also follow this pattern, although a more representative sample of species along a trophic gradient is needed to test this hypothesis.

### The gut microbiota in captive *versus* wild population of *A*. *burtoni*

Depletion of the gut microbial biodiversity in captivity has been now documented in several animal systems [53, 54]. Cichlids are no exception. The inbred strain of *A*. *burtoni* displayed a dramatic reduction of the natural microbial biodiversity (nearly 70% less diverse based on Chao1, Fig 1) and a profile (in terms of taxonomic content and phylogenetic diversity) characteristic of a distinct species when compared to conspecifics from a wild population. Except for three indicator OTUs uniquely found in this lab population (Table 3), this inbred strain did not host any significant additional taxon/OTU. Overall captivity simply resulted in a reduction of the microbial biodiversity. Responsible of this pattern might be the artificial food (flakes), which offers homogeneous and highly digestible material mainly constituted by proteins and fat, but poor in fiber with respect to a natural diet. Such reduction in fiber content, in particular, might cause the decreased microbial diversity observed in captive specimens, although the effect of a standard fish lab diet on the gut microbiota should be more formally investigated. Other factors might have concurred to this pattern; among others, presence of bactericides in the water tanks and recurring water changes in the aquarium, which progressively reduce exposure to the original bacterial pool.

It is nevertheless interesting to observe that most of the core taxa and all host-associated OTUs found in wild cichlids were also present in captive specimens, suggesting the existence of some host-specific effects in shaping the microbiota composition despite differences in environmental conditions and diet.

### The cichlid core gut microbiota

Presence of a gut microbial core, i.e. a shared microbial component among close host relatives, is indicative of inheritance and/or selectivity over a common set of microbial taxa, followed by a conserved plan for taxa retention and community assembling [11]. The existence of a species core, although still widely debated, has been now documented in several vertebrates, including humans [55], and fishes in captivity (e.g. in the rainbow trout [56], zebrafish [24]) and in the wild (e.g. trinidian guppies [27] and surgeonfishes [23]). More rarely presence and origin of a microbial core have been investigated across vertebrate species, remaining largely unexplored in the context of phylogenetically closely related species in nature [23, 53].

All cichlid species in this study, including the inbred laboratory strain, shared a small set of OTUs and a much larger set of bacterial taxa (Tables 1 and 2). The seven cichlid core phyla, as well as most core families and genera, are also typical associates of teleost fishes [22, 24] as well as of most vertebrates (including humans, [44]), proving this compositional core to be a signature of a vertebrate gut rather than cichlid-specific. Unlike most other freshwater fishes, however, all cichlid specimens in this study showed a relatively low abundance of Proteobacteria (Fig 2), which represent the typical primary component of the gut microbiota in freshwater fishes [21, 22, 31], including Midas cichlids from Nicaragua kept in laboratory conditions [34]. Examination of Proteobacteria representation in our two datasets (V12 and V34) indicates that we successfully amplified all five major lineages of this phylum (i.e. classes alpha to epsilon), thus excluding major primer biases and suggesting that African cichlids might carry a quite distinct microbial profile in terms of phyla relative abundance. We also note that here we have sampled entire gastrointestinal tracts (unlike in the majority of previous studies), thus providing a most comprehensive overview of the actual potential microbial biodiversity of the digestive tract. More sampling of wild fishes within and outside cichlids, however, are needed to corroborate this pattern.

At deeper bacteria taxonomic classification, the six cichlid species examined shared a relatively small proportion of core OTUs (1.4–1.8%), probably larger with an increase in sequencing effort. Retention of this small core of bacteria, also in captivity, might underline an important role in the cichlid “holobiont” system. Among the core OTUs that were identified as host-associated according to best BLASTN hits, most had high similarity to gut microbes of other fishes outside Lake Tanganyika (Table 1), implying promiscuity of some gut microbes beyond cichlids and lake boundaries, although with a certain specificity to fish hosts. OTUs that are apparently fish-specific have been also recently detected in trinidadian guppies [27], suggesting the existence of a putative fish core microbiome. Although an interesting scenario, caution should be taken considering the current ambiguous classification of many bacteria as either host or environmental associates in present databases [48, 49].

The three most abundant core OTUs in cichlids were classified as *C*. *somerae*, *P*. *shigelloides*, and *C*. *perfringens*. All the three species have been previously associated to the intestinal tract of freshwater fishes [24, 27, 57], suggesting a tight link to the fish biology. *C*. *somerae*, in particular, an obligate anaerobe putatively involved in the metabolism of vitamin B12 [57], was recently found as a core species in three farm fishes [58], and in the trinidian guppy *Poecilia reticulata* [27]) and it is certainly worthy of further investigation.

When we looked at cichlid intraspecific level, the proportion of core OTUs shared among conspecifics was rather small (13–15% on average) despite including some of the most abundant sequences found in all fishes. In another study surveying wild surgeonfishes, a similar microbial core pattern was detected, suggesting the existence of a small but stable microbial component in individual fish species. Such cichlid intraspecific core was typically larger than expected by chance (i.e. when compared to a random sampling of individuals) and significantly larger in host intraspecific than interspecific pairwise comparisons. Additionally, several of these species core OTUs were shared across multiple cichlid species. Altogether this suggests that the transfer of core OTUs among conspecifics is preferential, but not exclusive, and diminishes with increasing host phylogenetic distance. This pattern might be, at least in part, favoured by a certain level of vertical transmission of gut microbes through host generations, as now reported in several vertebrates [14]. In cichlids, mouthbrooding, i.e. the buccal incubation of the eggs until development to larvae, increases contact among conspecifics during the development of an intestinal microbiota and might favour the recruitment of bacteria from a common microbial pool; indeed, all species in this study are mouthbrooders. Future comparisons between mouthbrooding and substrate spawning cichlids, as well as mouth-only sequencing, could assess the actual influence of parental care strategies in favouring microbiota transmission.

In laboratory reared specimens, sharing of the same tank can certainly facilitate horizontal transmission of gut microbes; nonetheless the proportion of core OTUs in the laboratory specimens was smaller than that among wild conspecifics and the smallest when compared to all other wild species according to V34 (Fig 3), pointing to rearing conditions (including diet) as determinant in this case.

Overall, our findings represent an important preliminary characterization of the cichlid microbiota. Several other factors, such as population structure, geography and gender, need to be considered and might account for some similarities/differences observed in the microbial composition of these fishes. A much deep and large-scale investigation of the gut microbiota in cichlids is also necessary. In particular, we now need to characterize the phylogenetic and functional microbiota profiles from a broad range of wild cichlids along their trophic gradient; these data can be very valuable to inform on the dynamics of the microbial communities in relation to the host trophic niches and phylogenetic relationships. Altogether, these findings will serve to explore the fascinating role of these microbial consortia in the process of cichlid ecological diversification.

## Supporting Information

S1 Fig Schematic representation of the phylogenetic relationships among the five Perissodini species according to [42]. (PDF)

S2 Fig Rarefaction curves of the observed OTUs for each specimen (single curves), from V12 (a) and V34 (b). Perecc curve (highlighted with a *) is shown in the same graph with Permic. The two 16S fragments returned highly similar patterns of OTUs richness, with Astbur displaying the most diverse microbiota of all cichlids, still far from saturation. For Perissodini species the sampling effort was likely sufficient to recover most of their microbiota diversity (with the exception of one *Plestr* individual).
(PDF)

S3 Fig Unweighted Unifrac UPGMA trees of V12 (a) and V34 (b) based on a Jackknife analysis with 100 repetitions at 3386 and 4148 sequences per sample, respectively. (PDF)

S4 Fig Maximum Likelihood trees (100 bootstrap replicates) of Cyanobacteria. Cichlids OTUs are shown as individual numbers at the branch tips together with representative sequences downloaded from the nt database (shown with AccNos) from the three major groups: Cyanobacteria (in black), Environmental Melainabacteria (in pink) and Gut Melainabacteria (in red) (as classified by [49]). The exact branch separating gut from environmental Melainabacteria is only putative. Sequences were aligned with Infernal in the RDP pipeline and a tree built with PhyML. Ten out of 81 OTUs in V12 and six out of 45 OTUs in V34 belong to the gut Melainabacteria clade and were exclusively found in *Astbur*.
(PDF)

S1 Table Cichlid samples information. (XLS)

S2 Table Libraries and primers information. (XLSX)

S3 Table Sequence data summary after quality filtering, singletons and Cyanobacteria removal. (XLSX)

S4 Table Best BLASTN hits of the 28 cichlids core OTUs. (XLSX)

S5 Table Significant Indicator Taxa in comparisons across host species, genera and tribes. (XLSX)

S6 Table Significant Indicator OTUs (value >0.6, p<0.01) in comparisons across host species. (XLSX)

S7 Table Significant Indicator OTUs in comparisons between tribes. (XLSX)
