# Supplementary figures and images for: Gut Microbiota Dynamics during Dietary Shift in Eastern African Cichlid Fishes
Source: PLoS One. 2015 May 15;10(5):e0127462. doi: 10.1371/journal.pone.0127462 (PMC4433246; doi:10.1371/journal.pone.0127462)

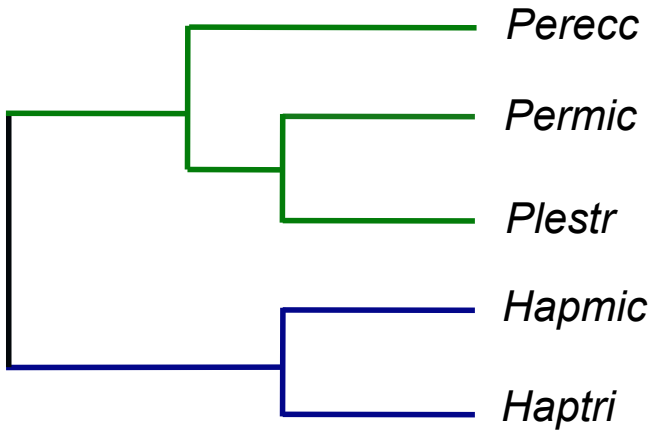

Supplement: S1 Fig — (PDF) [file pone.0127462.s001.pdf]

a)

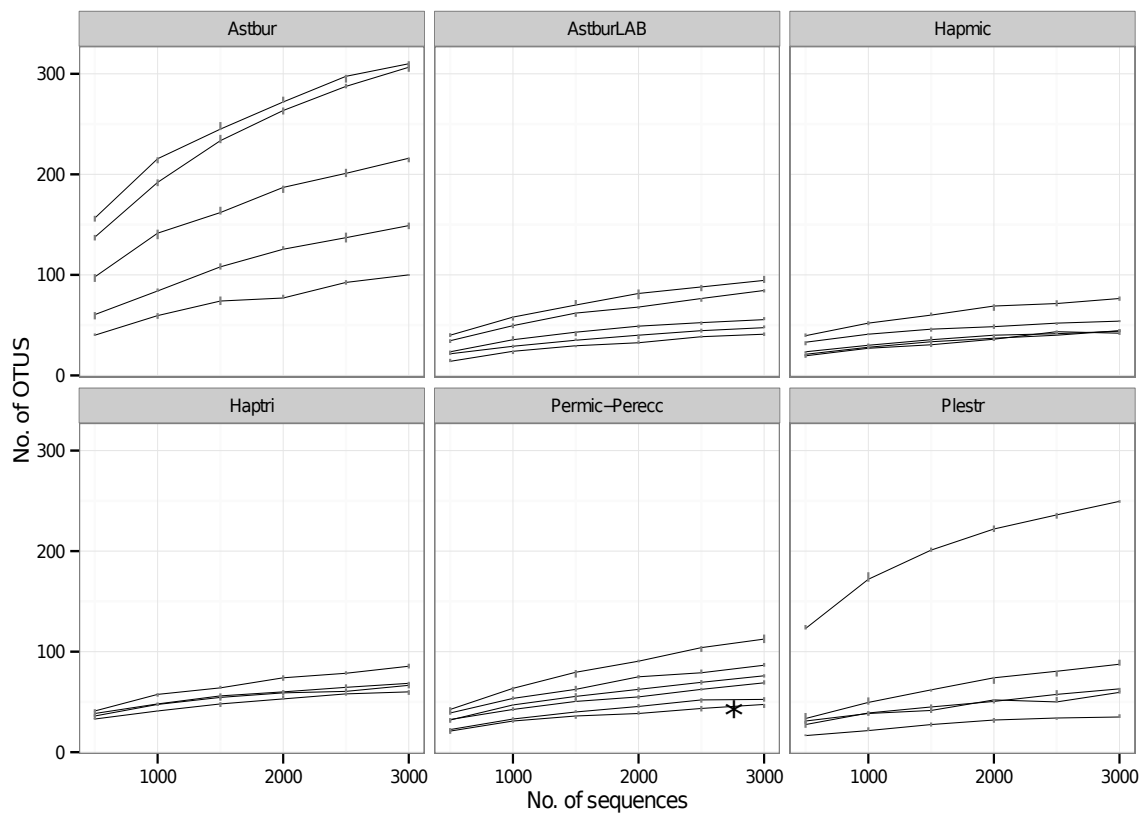

b)

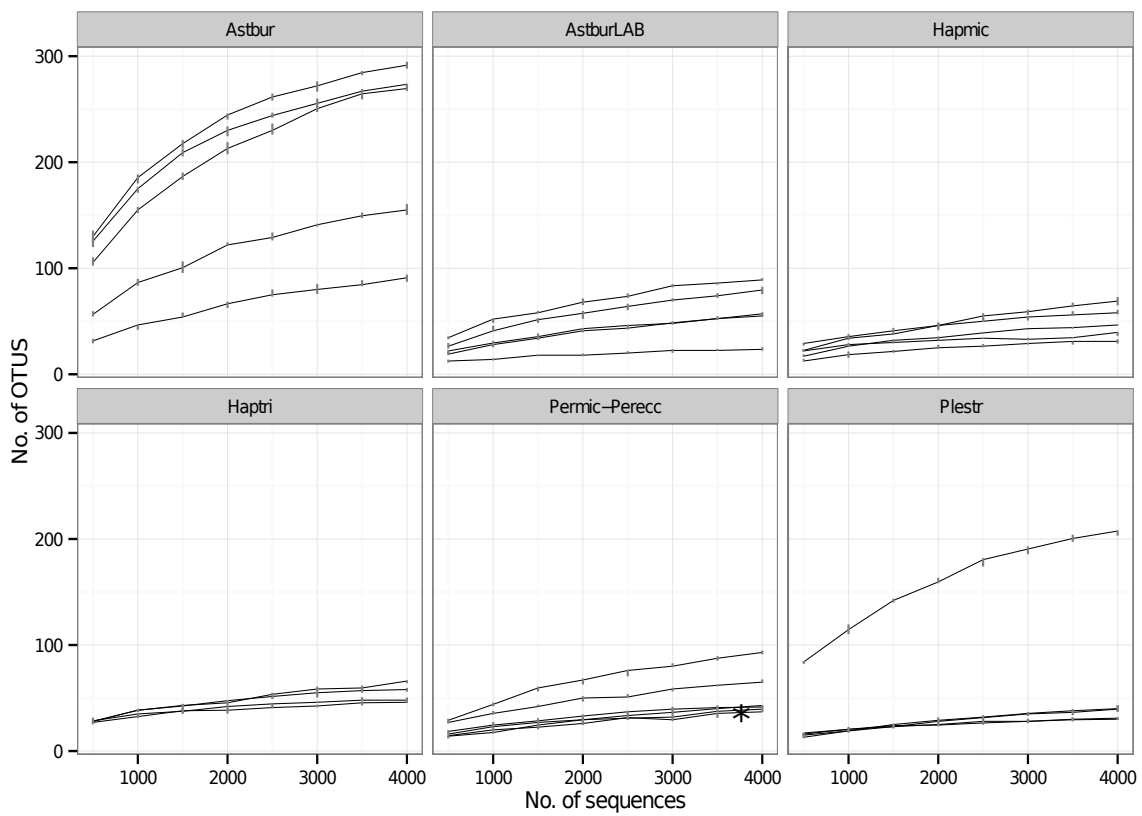

Supplement: S2 Fig — Perecc curve (highlighted with a *) is shown in the same graph with Permic. The two 16S fragments returned highly similar patterns of OTUs richness, with Astbur displaying the most diverse microbiota of all cichlids, still far from saturation. For Perissodini species the sampling effort was likely sufficient to recover most of their microbiota diversity (with the exception of one Plestr individual). (PDF) [file pone.0127462.s002.pdf]

# V12

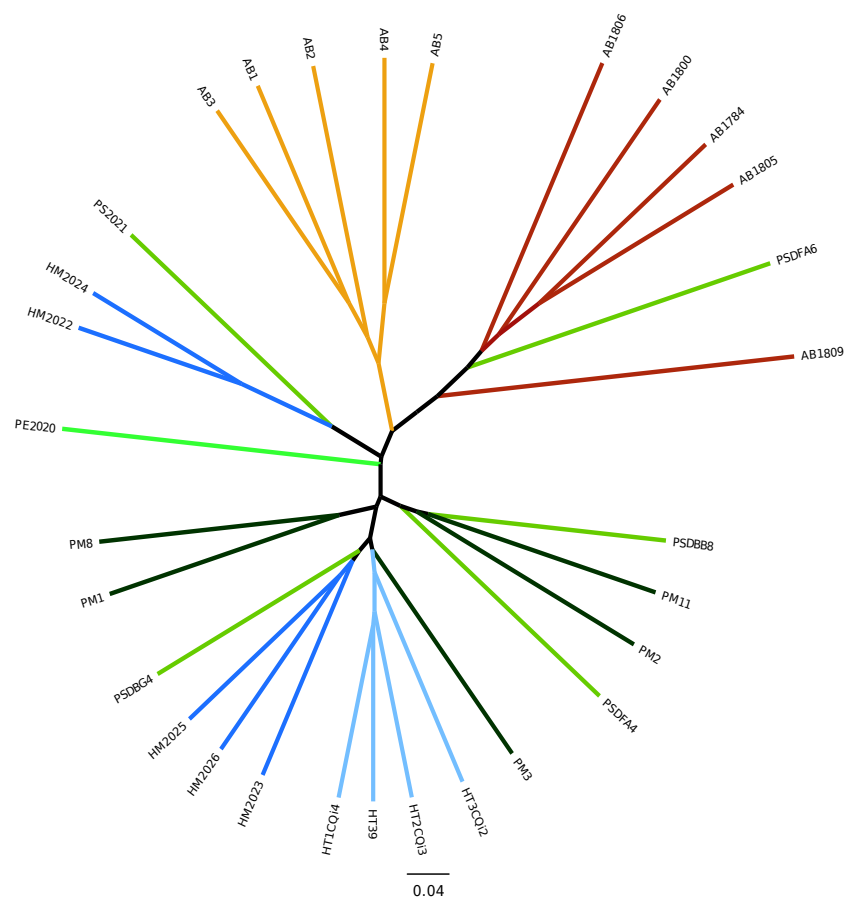

# V34

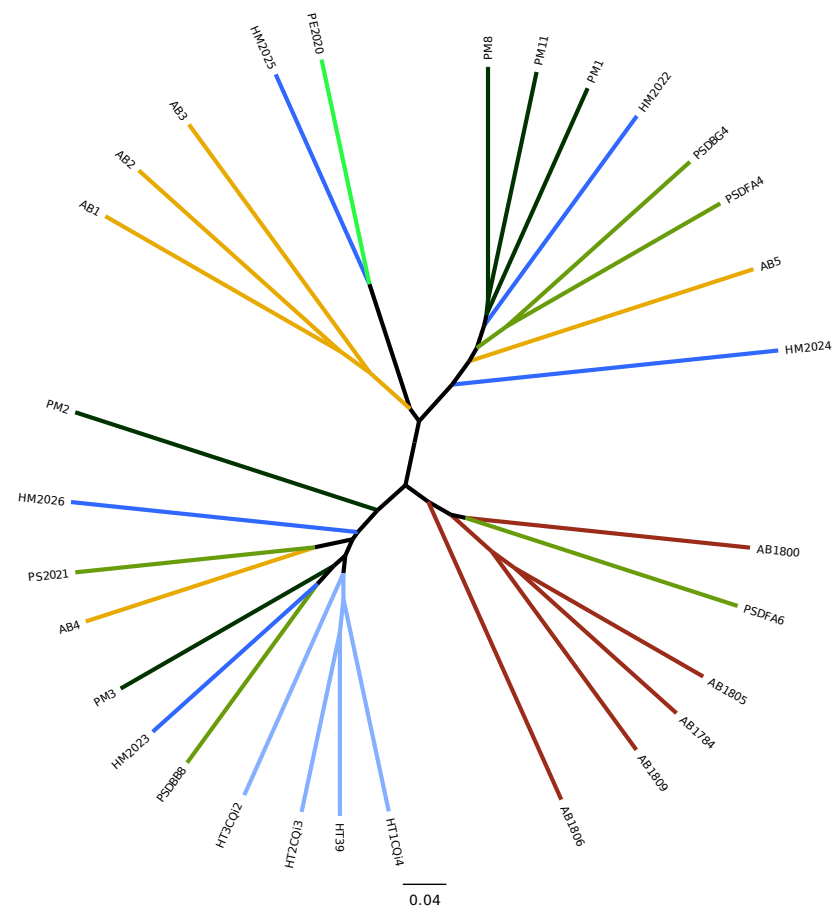

Supplement: S3 Fig — (PDF) [file pone.0127462.s003.pdf]

a) V12

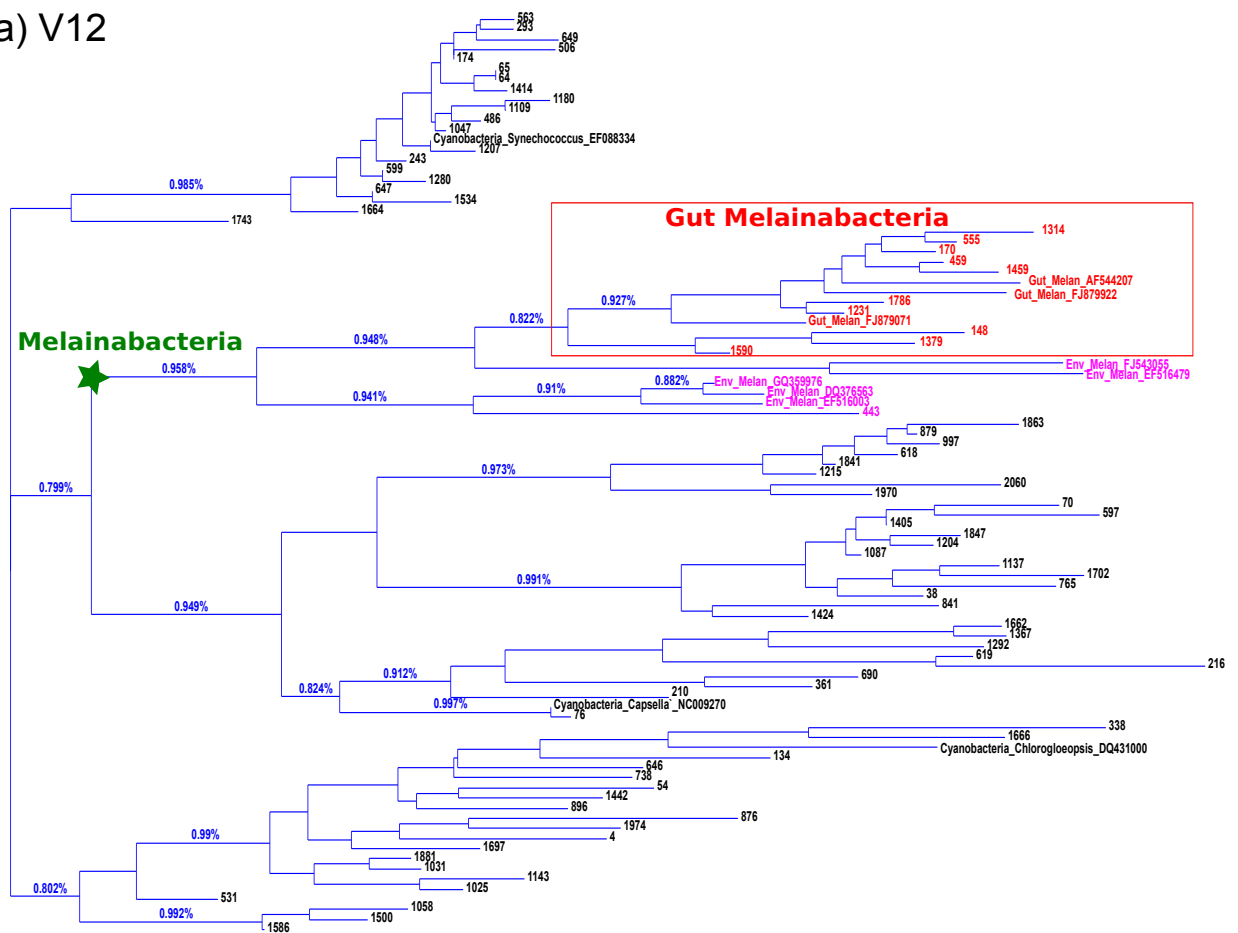

b) V34

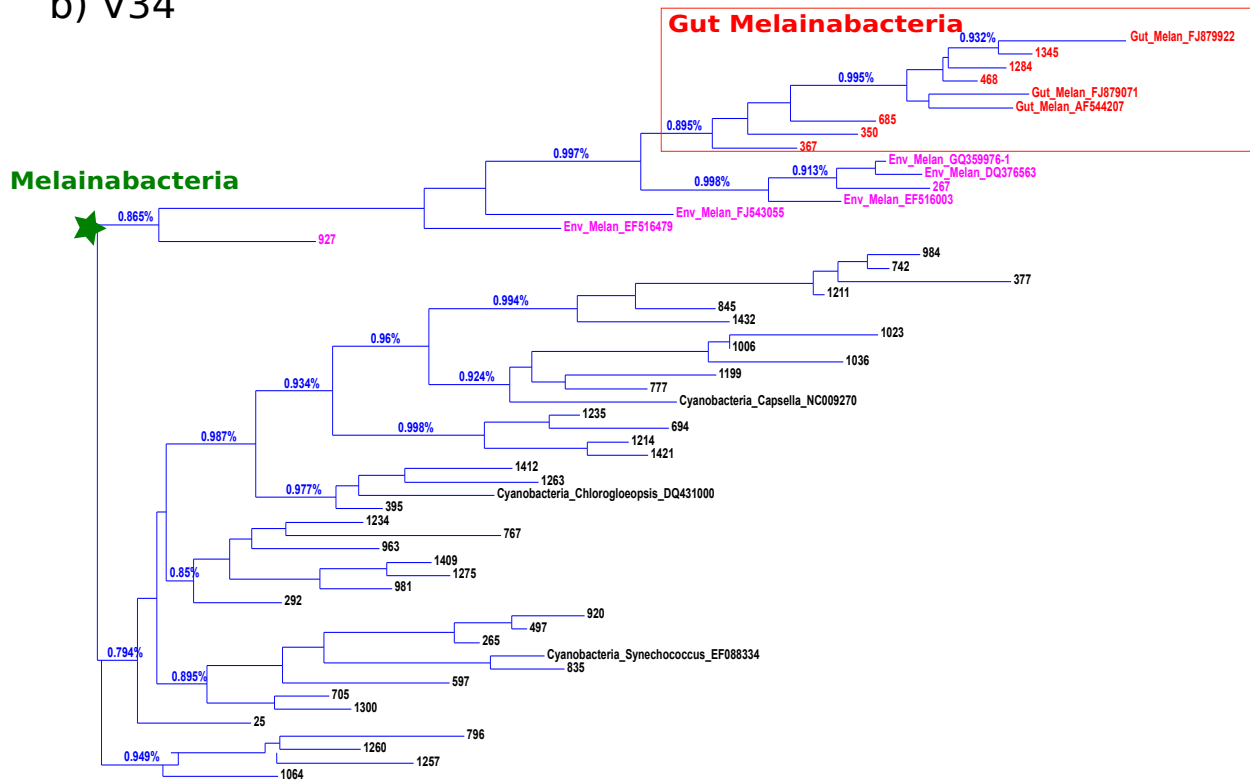

Supplement: S4 Fig — Cichlids OTUs are shown as individual numbers at the branch tips together with representative sequences downloaded from the nt database (shown with AccNos) from the three major groups: Cyanobacteria (in black), Environmental Melainabacteria (in pink) and Gut Melainabacteria (in red) (as classified by [49]). The exact branch separating gut from environmental Melainabacteria is only putative. Sequences were aligned with Infernal in the RDP pipeline and a tree built with PhyML. Ten out of 81 OTUs in V12 and six out of 45 OTUs in V34 belong to the gut Melainabacteria clade and were exclusively found in Astbur. (PDF) [file pone.0127462.s004.pdf]
